# Linear IgA and IgG bullous dermatosis*

**DOI:** 10.1590/abd1806-4841.20164630

**Published:** 2016

**Authors:** Karina de Almeida Pinto Fernandes, Kely Hernández Galvis, Anndressa Camillo da Matta Setubal Gomes, Osvania Maris Nogueira, Paulo Antônio Oldani Felix, Thiago Jeunon de Sousa Vargas

**Affiliations:** 1 Hospital Naval Marcílio Dias (HNMD) - Rio de Janeiro (RJ), Brazil; 2 Hospital Federal dos Servidores do Estado (HFSE) - Rio de Janeiro (RJ), Brazil; 3 Hospital Federal de Bonsucesso (HFB) -Rio de Janeiro (RJ), Brazil

**Keywords:** Basement membrane, Immunoglobulin A, Linear IgA bullous dermatosis

## Abstract

Childhood linear immunoglobulin A dermatosis is a rare autoimmune vesiculobullous disease. It results in linear deposition of autoantibodies (immunoglobulin A) against antigens in the basal membrane zone, leading to subepidermal cleavage. Additional depositions of immunoglobulin G and complement-3 might occur. It is still debated whether concomitant findings of immunoglobulins A and G should be considered a subtype of this dermatosis or a new entity. Further studies are needed to recognize this clinical variant.

## INTRODUCTION

Chronic bullous dermatosis of childhood, or linear immunoglobulin A bullous dermatosis (LAD) of childhood, is a rare, autoimmune subepidermal bullous disease. It is characterized by tense blisters, usually on an erythematous base, usually in the perineum and perioral regions. The string of beads sign is characteristic when new lesions appear around the previous ones.^1,2^ Mucosal lesions can also be affected, especially in the oral and ocular regions. Oral lesions may be painful ulcers and even desquamative gingivitis. Chronic conjunctivitis, synechiae formation, and blindness might occur. Pharyngolaryngeal mucosa may also be affected, which may lead to respiratory difficulty.^3^ The disease develops after six months of age, and shows incidence peaks in preschool children. Spontaneous remission might occur within two years, or it may persist until puberty.^2,3^ The pattern of the mucosal lesions is similar to patients with cicatricial pemphigoid (evolution with scars), and might be explained by epitopes extending to the carboxyterminal portion of the 180 kDa bullous pemphigoid antigen (BP 180).^4^ Its pathogenesis is unknown. HLA-B8, -DR3, and -DQ2 rates increase in these patients.^2^ Some disease triggers reported include drugs (vancomycin, lithium, phenytoin, furosemide, captopril), infections, autoimmune diseases (post-streptococcal glomerulonephritis and inflammatory bowel disease, particularly ulcerative colitis), and lymphoproliferative disorders.^5,6^

## CASE REPORT

**Figure 1:**
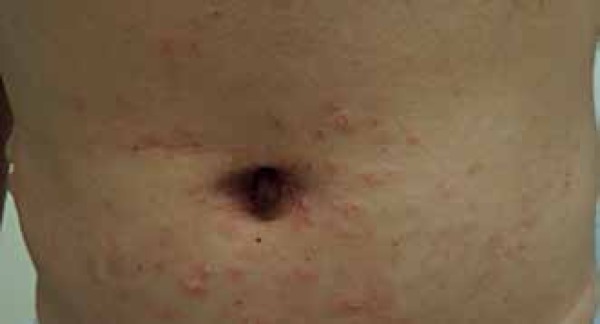
Well-demarcated erythematous papules on the abdomen

**Figure 2:**
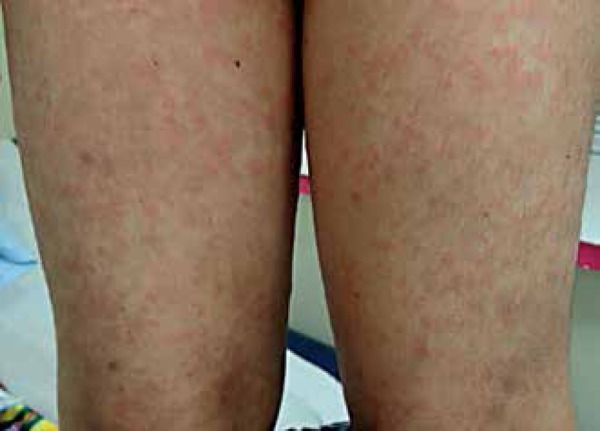
Well-demarcated erythematous papules on the lower

**Figure 3:**
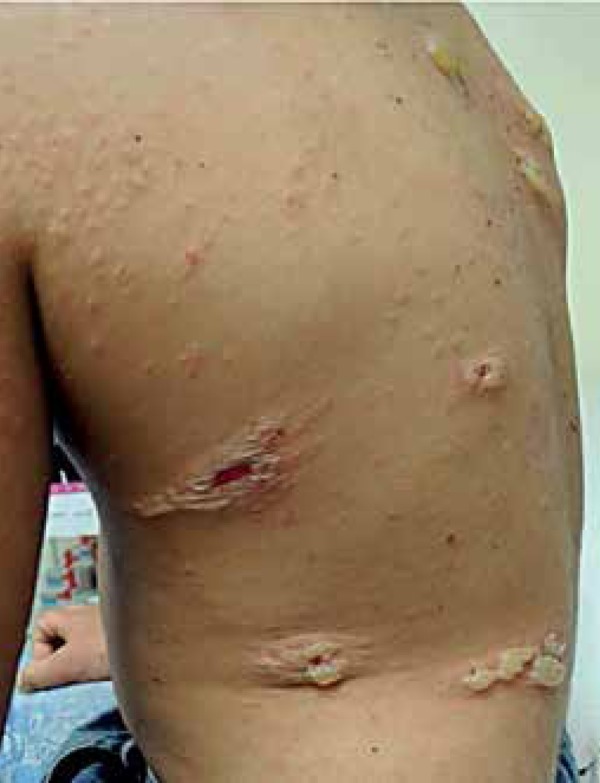
Tense bullous lesions with purulent content, some of which around old lesions, displaying the string of beads sign on the back

**Figure 4:**
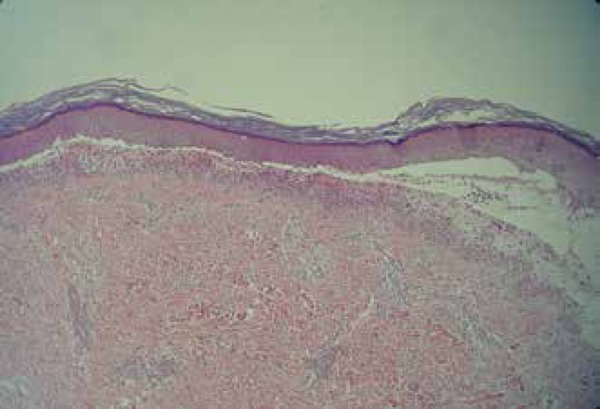
Histopathological examination showing subepidermal blister formation and inflammatory infiltrate, with predominance of neutrophils spread in band pattern along the dermoepidermal junction (Hematoxylin - eosin x100)

**Figure 5:**
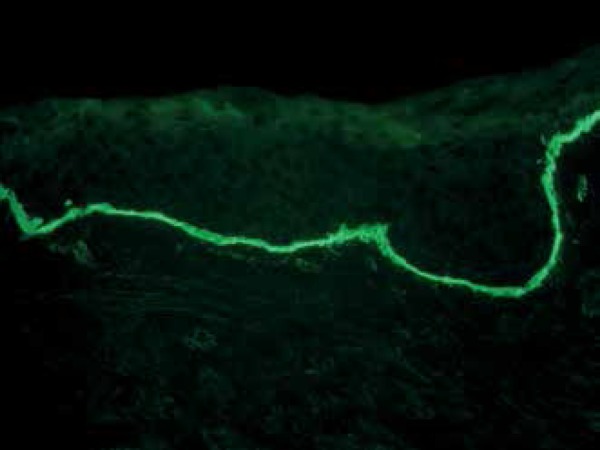
Direct immunofluorescence of skin with anti-IgG antibody showing high-intensity, linear patterns along the basal membrane

**Figure 6:**
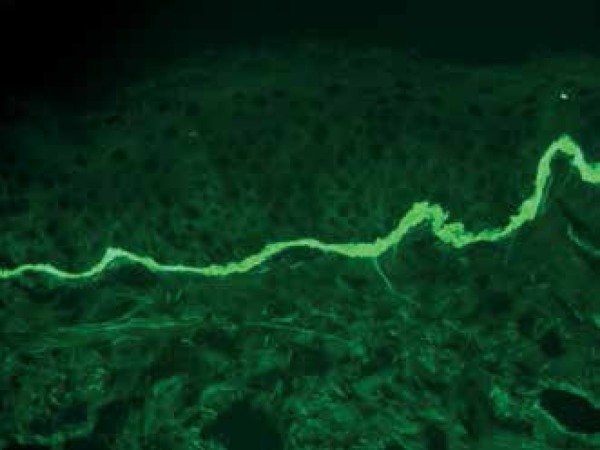
Direct immunofluorescence of skin with anti-IgA antibody showing high-intensity, linear patterns along the basal membrane

A seven-year male patient sought medical attention complaining of widespread papules and blisters on the back after two months. Examination found well-demarcated erythematous papules on his abdomen and lower limbs, as well as tense bullous lesions with purulent content. Some of which were around old lesions, displaying the string of beads sign on his back (Figures 1 to 3). Laboratory tests showed high rates of leukocytosis, erythrocyte sedimentation, and C-reactive protein. Serology for antiendomysium and transglutaminase was negative, and glucose-6-phosphate dehydrogenase (G6PD) showed no alterations. Skin biopsy and direct immunofluorescence (DIF) testing was performed. Histopathological examination showed subepidermal blister formation and inflammatory infiltrate, with predominance of neutrophils spread in band pattern along the dermoepidermal junction (Figure 4). DIF testing showed linear deposition of Immunoglobulin A (IgA) and Immunoglobulin G (IgG) along the basal membrane, confirming the diagnosis of linear IgA and IgG bullous dermatosis (Figures 5 and 6). The patient was admitted for the treatment of secondary infection of the lesions. Dapsone 0.5mg/kg/day improved his skin condition. As the evolution showed repeating conjunctivitis, oral prednisolone 0.5mg/kg/day and corticosteroid eye drops were used. We increased dapsone dose to 2mg/kg/day. Despite the clinical control, the patient showed eyelid adhesion, which was surgically corrected. The patient is currently being followed up by dermatologists and ophthalmologists.

## DISCUSSION

LAD of children must be differentiated from dermatitis herpetiformis and bullous pemphigoid of childhood, as they share similar clinical and histopathological characteristics. Direct immunofluorescence (DIF) is essential for its correct diagnosis.^1,2^ DIF shows linear and homogeneous IgA deposition in the basal membrane zone (BMZ), but IgG (up to 25% of cases) and C3 can be detected.^3-5^ The main target antigens are the 97 and 120 kDa extracellular domains of BP 180 (collagen XVII). However, others have been reported, such as collagen VII, bullous pemphigoid 230 kDa antigen, and laminina.^7^ The term linear IgA and IgG dermatosis (LAGD) is proposed for a subtype or variant of the disease that occurs with deposition of both immunoglobulins and that is found more in adults than in children.^7^ A study of four patients with IgA and IgG deposition in the BMZ concluded that the clinical and histopathological findings, as well as the target-antigen (97 kDa extracellular domain of BP 180), were similar to patients with LAD.^8^ For some authors, LAGD and childhood LAD share similar characteristics and are manifested as a bullous, pruritic rash.^9^ Dapsone is the most common drug in the treatment of this disease. However, it should be used with care, due to the risk of side effects, which include: hemolysis and methemoglobinemia (which are dose-dependent); motor neuropathy; neutropenia; and hepatitis.^10^ Therefore, patient's blood count must be regularly moniterd, as well as their reticulocyte, haptoglobin, methemoglobin, and liver enzyme rates.^5,10^ Before the treatment begins, G6PD enzyme levels should be assessed, as any related dysfunctions contraindicate their use.^5^ The initial drug dosage should be 1-2mg/kg/day, up to 3-4mg/kg/day, according to the patient's clinical response and tolerance.^5,10^ Cases with IgA and IgG deposition might require additional treatment with systemic corticosteroids.^1^ Difficult cases might require immunosuppressants, such as azathioprine, mycophenolate mofetil, and cyclosporine. The use of antibiotics such as erythromycin and dicloxacillin have been reported in mild cases.^2,5^ Once the disease is controlled, the minimum dose of medication is required to control the symptoms.^10^
